# Innovations in healthcare delivery: Human papilloma virus self sampling diagnostics and participatory innovations for CCS

**DOI:** 10.1002/cam4.6201

**Published:** 2023-06-16

**Authors:** Evans Appiah Osei

**Affiliations:** ^1^ Purdue University West Lafayette Indiana USA

**Keywords:** participatory, innovations, cervical cancer screening, HerSwab, human papilloma virus, innovations

## Abstract

**Background:**

Human papillomavirus (HPV) infection is a major contributor to the development of cervical cancer, resulting in over 500,000 cases and 266,000 deaths annually worldwide. Previous cervical cancer screening programs have been successful in reducing cervical cancer rates, but have faced challenges such as low acceptance and adherence rates. Innovations in screening technology, such as the HerSwab self‐sampling test, have the potential to increase awareness, acceptance, and participation in cervical cancer screening programs.

**Aim:**

This literature review examines the effectiveness of HerSwab and participatory innovations in increasing adherence to cervical cancer screening.

**Method:**

This manuscript comprised a comprehensive narrative literature review encompassing the years 2006–2022. The review process adhered to the PRISMA diagram as a guiding framework. Among the search terms utilized, a total of 200 articles were initially retrieved. However, after applying the predefined inclusion criteria, only 57 articles were included.

**Results:**

The HerSwab self‐sampling test is described, including how it is performed, challenges, and facilitators, and evaluation and assessment of its effectiveness. While the HerSwab diagnostic test is not currently widely available, studies should assess its feasibility in less developed countries where cervical cancer mortality rates are high.

**Conclusion:**

By increasing awareness and availability of innovative screening techniques, such as HerSwab, we can work toward reducing the incidence of cervical cancer and improving outcomes for women worldwide.

## 1 INTRODUCTION

Human papillomavirus (HPV) infection causes approximately 528,000 cervical cancer (CC) cases, and 266,000 mortalities yearly worldwide.^1^ Based on this, the Eve Medical company in Canada designed a cervical cancer diagnostic kit known as the HerSwab at‐home kit which has been approved in Canada (MDL license 94847).^2^, ^3^ HerSwab diagnostic test is a tampon‐shaped kit used by women to collect their vaginal sample which is then mailed to the hospital for analysis. HerSwab is approved for use in Canada and Europe for diagnosing CC and other Sexually Transmitted Infections (STIs) such as chlamydia and gonorrhea.^4^ Nevertheless, this paper focuses on innovations in Human papillomavirus diagnostics (HerSwab and other HPV self‐sampling methods) and participatory innovations to help increase cervical cancer screening uptake.

Participatory innovations have been explained by other researchers as the process where end users of a product are allowed to take part in the designing, and evaluation of a product to enhance patronage and convenience with use.^5^ This is where data feedback is collected from users of a service at the design stage.^6^ Recently, an author identified that successful participation (uptake and adoption) in cervical cancer screening services especially in low and middle‐income countries has not been extensively studied.^7^ They added that the views and opinions of users (females) are often ignored when designing these screening strategies which could lead to poor uptake and acceptance.

Several studies have been conducted among healthcare providers to identify effective strategies for improving CC screening uptake, while others have explored the role of community partnerships and engagement in motivating women to undergo screening.^7^, ^8^, ^9^, ^10^, ^11^ To improve the acceptance and reduce potential rejection of HerSwab (Home‐based HPV test kits), it is recommended that innovative approaches, such as community participation, healthcare worker involvement, women's engagement, and stakeholder involvement, be employed during the development, and testing phases.

Engaging women as the end‐users is critical in ensuring that the test meets their needs and preferences. Their inputs are valuable in the development process of HerSwab, which can result in a screening tool that is more acceptable and user‐friendly for women. In addition to women, involving their relatives and significant others is also important as they can influence women's decision to participate in screening practices. Their support and encouragement can help overcome potential barriers and increase the uptake of HerSwab. Overall, engaging these stakeholders is essential for ensuring the success of HerSwab as a cervical cancer screening tool.

The worldwide statistics for cervical cancer in the year 2018 was 569,847 and out of this number, more than half of the women diagnosed with CC (311,365) could not survive it.^12^ There was a 10% increase in the expenditure made on cancer alone in the USA from $190.2 billion in 2015 to $208.9 billion in 2020 of which cervical cancer is not an exemption.^13^ Moreover, the National Cancer Institute documented that in 2019, roughly 295,382 women from the USA were diagnosed with CC while the American Cancer Society in 2022 gave an estimate of 14,100 new invasive CC cases and nearly 4280 mortalities were recorded in the US.^14^

Based on this, several diagnostic tests and treatment strategies have been implemented to help reduce the mortalities and morbidities caused by CC worldwide.^15^ The screening services for CC, especially the Papanicolaou (Pap) test was initiated in the 1940s while others were introduced in the 1950s which has led to a significant decline in the number of CC cases and deaths most especially in the mid‐20th century.^16^, ^17^ The test is conducted by placing a speculum through the vagina of a woman to access the cervix.^18^ A brush is then used to take samples (cells) from the cervix for analysis which may help detect the presence or absence of the HPV and early cervical lesions.^19^ Evidence suggests that the screening should begin at 21 years, performed every 3 years till a woman turns 30.^20^ Following 30 years Pap test and HPV tests are to be done concurrently every 5 years.

The Pap test and HPV test were recommended by the American Cancer Society, the American College of Obstetrics and Gynecologists, and the United States Preventive Service Taskforce to be effective in the early detection of cervical cancer.^21^ Cervical cancer screening test (Pap Smear and HPV test) has resulted in a significant reduction of CC incidence in both developed and developing countries^22^, ^23^, ^24^ with a recorded number of 50 per 100,000 cases annually in some African countries (Malawi, Mozambique, Comoros, Zambia, Zimbabwe, and Tanzania), 5.5 per 100,000 in Australia and 6.6 per 100,000 in the United States of America.^25^ It was further reported by other authors that with effective and innovative cervical cancer screening could reduce the incidence of cervical cancer to as low as <4 per 100,000 women by 2100.^26^

Despite the benefits associated with cervical cancer screening, it has faced several challenges including reluctance to partake in CCS, high cost of screening services, unnecessary delays during screening, discomfort during screening, and concerns about invasion of privacy; all of which decrease CCS patronage by women.^27^ Hence, this current paper presents innovations in CC diagnostics and participatory innovations to help increase screening uptake for early detection and prevention of CC.

## 2 METHODS

**FIGURE 1:**
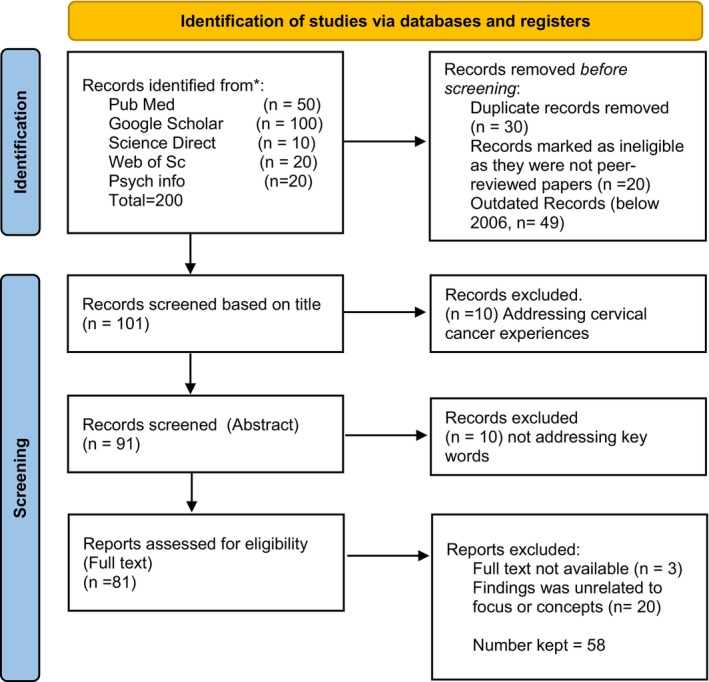
PRISMA flow diagram for study inclusion.

Keywords used in searching for literature included cervical cancer screening, HPV, HerSwab, diagnostic innovations, and participatory innovations. Search engines used were PubMed, Google Scholar, Science Direct, Web of Science, and Psych Info. The year range was 2006–2022 with most of the studies 39 (70%) published between 2017 and 2022. The eligibility criteria were studies published in English between 2006 and 2022, addressing CCS, HPV, HerSwab, and other self‐sampling diagnostic tests and participatory innovations. The search terms retrieved 200 articles, however only 58 were included based on the inclusion criteria. Those that were found on treatment, experiences with cancer, cervical cancer descriptions, etc were eliminated (Figure 1).

## 3 DESCRIPTION OF HERSWAB SELF SAMPLING KIT

Several innovative CC diagnostic tests have been found in the literature including dry flocked swab (DF), Delphi Screener and a wet dacron swab (WD), or a HerSwab (HS) and Qvintip (QT) device as well as self‐sample HPV urine test kits (Colli‐Pee device).^29^, ^30^ Notable among these tests was an innovative diagnostic test that has been recently developed by Eve Medical to help overcome the barriers identified. The test is known as HerSwab at‐home diagnostic kit where women can take their samples in their homes using a tampon or a brush and send them either directly or by mail to the hospital.^2^ Women are taught to stand with their legs apart and insert the tampon into the vagina for one to 2 h, before removing it.^31^, ^32^ The tampon is then placed in a 30‐mL sample bottle containing 10 mL of saline solution to prevent drying of the sample which is then mailed or sent directly to the health care facility. This test is different from the physician HPV sampling because the sample is taken by the woman herself without having to expose their nakedness to anyone. This method does not involve the use of a speculum thereby helping to reduce the fear associated with the discomfort of speculum insertion.

Eve Medical Inc. is a profit social enterprise in Canada (Toronto) founded in 2010 and is an accredited (ISO13485) medical device manufacturer that helps in designing home‐based test kits (HerSwab) for the detection of HPV and other STIs.^3^ The HerSwab test kit, aside from helping in the early detection of precancerous lesions, is effective as a hospital‐based HPV test.^4^, ^32^ El‐Zein et al.^4^ added that it is more preferred and comfortable for most women than hospital‐based HPV testing. Participants of a study who self‐collected their sample using the HerSwab kit reported that it was easy (97.1%) and comfortable (88.3%).^32^ The sample after collection is mailed the same day or transported to the health care facility the same day to ensure the accuracy of the results. Even though the cost for HerSwab self‐sampling test could not be identified, the cost for other self‐sampling HPV tests in the US such as the Nurx kit and EverlyWell kit costs US$49 to US$79 and US$24.99 to $49.10, respectively.^33^ With regard to HerSwab effectiveness compared to hospital‐based HPV diagnosis, in the identification of HPV, some authors discovered that there was not much difference (4.8 difference) in HPV detection sensitivity among home‐based self‐sampling tests; HerSwab (87.6%) and hospital‐based HPV testing (92.4%).^4^ However, HerSwab is innovative because samples are self‐collected, done at home, easy to do, and involves less discomfort as no speculum is used.^4^, ^34^ It was also worth noting from a study among women from rural areas in Guatemala that women with lower social socioeconomic status and poor health‐seeking behaviors were highly interested in this innovative method of screening.^34^ HerSwab HPV method is innovative because results could be sent by mail to women without them going to cue at the health care facility to wait for their results.

Furthermore, a survey in 2019 a cross‐sectional study conducted in Canada among 1217 women ascertained that close to half of the women 329 (47%) collected their own samples at home for testing using HerSwab Kits.^35^ To improve cervical cancer screening uptake and reduce cervical cancer incidence worldwide, the WHO recommended that models using self‐sampling by women in the community should be promoted and encouraged.^11^ Other authors also reported that championing and implementing this self‐sampling testing system for CC could broaden the screening coverage and help limit embarrassment, and discomfort experienced by women as a result of physician sampling collection.^33^

## 4 CHALLENGES AND FACILITATORS OF INNOVATIONS IN CERVICAL CANCER DIAGNOSTICS AND PARTICIPATORY INNOVATIONS

The willingness to undergo the CCS test was reported to double in women collecting their samples as compared to samples collected by physicians.^36^ Motivators for self‐sampling HPV tests at home included having adequate knowledge about the self‐sampling method, and thinking that cervical cancer is serious.^37^, ^38^ Another facilitator was the absence of encounters with physicians during the test.^39^ Other women also perceived the collection process of self‐HPV samples to be simple compared to the hospital HPV sampling.^40^

Even though this innovative test has numerous benefits for women worldwide, there are some anticipated and unanticipated challenges. For instance, a recent study discovered that this test has not been approved by the FDA in some developed countries such as the USA.^41^ Moreover, it was reported that the willingness to collect home‐based cervical swaps using HerSwabs decreased slightly from 76.6% to 63.4% following their first sampling collection even though they gave positive comments about it.^29^ However, the reason for the decline in interest was not clear; hence the need for further studies to assess concerns with these innovative diagnostic tests.

Moreover, results comparing the sensitivity of physician HPV sampling to self‐sampling by women have revealed a slightly decreased sensitivity in detecting cancer lesions with self‐collected samples.^37^, ^42^ A survey conducted in the United States also found that the perception of collecting results correctly to get accurate results was what most women were bothered about.^37^ The mailing of results was something that participants of some studies were concerned about due to the possibility of a breach of privacy and confidentiality.^33^, ^39^

Even though most studies have mentioned the significance of HPV self‐sampling as a motivator for women unwilling to undertake HPV testing at the hospital,^33^ it is still not widely used worldwide, especially in low‐middle‐income countries. Several factors could be attributed to the reason why HerSwab is not accepted in developing and low‐income countries. It is recommended that current studies focus more on the implementation and sustainability of HPV Self‐sampling (HerSwab) to help reduce CC burden and incidence. Recently, several efforts are geared toward cervical cancer screening, and treatment.^43^, ^44^ Nevertheless, awareness of HerSwab and other self‐sampling methods is inadequate which could lead to poor acceptance and adherence to the screening schedule by women worldwide.

Some unanticipated challenges are expected especially in LMIC such as difficulty taking samples by the women, storage of the samples after collection, and transportation of the samples to the health center. It is therefore necessary for healthcare professionals to heighten education on the test and the process of doing it on various social media platforms to help solve some of these unanticipated challenges. Even though the cost of HerSwab HPV kits is not known publicly, it is anticipated that some women especially those in less developed countries may find it a challenge to purchase these kits. A similar self‐sampling HPV screening kit (The Nurx kit) costs $49.^33^ Not only will women find it difficult to afford, but individuals may reuse them due to the high cost, which may predispose them to vaginal infections. Previous studies mentioned that the results could be mailed or sent as text messages.^41^ However, it will be a challenge for women in low‐resource countries where the mailbox is not available in most homes and some women do not have personal phones.

Some suggestions to help overcome these anticipated challenges could be to make test kits free and available and accessible to all women in both developed and developing countries. Short videos could be developed on how to self‐collect samples for HPV and disseminated to women worldwide on their phones to help overcome the perceived difficulty in HPV collection. Community health nurses should be educated on these new innovative test kits, and their usage to help train women on how to take samples following physicians' orders. Instituting measures to follow‐up on those with positive results and treating them could also influence the decision of other women to embrace this new strategy.^41^ It is also important that the FDA in the US and other developed and developing countries assess this strategy for future consideration and improvement to help reduce the incidence rate of CC.

## 5 EVALUATION OF HERSWAB HPV SELF‐SAMPLING TECHNIQUE

Evidence suggests that the implementation and utilization of the HerSwab sampling test will be successful in increasing CCS patronage, early detection of CC lesions, and reducing mortalities associated with CC.^34^, ^42^, ^43^ For instance, a study conducted among women in New Zealand on HPV self‐sampling acceptability reported that 78% indicated a preference for self‐sample, compared to 22% who preferred a physician‐collected sample.^29^ Furthermore, research done in Canada among under‐screened women from Rural Ontario discovered that women who received HPV self‐sampling kits were 3.7 times more likely to screen than Physician collected samples.^47^

More so, studies comparing the acceptability of self‐sampling using HerSwab to physician‐collected samples detected an increased preference for HerSwab for hospital‐based HPV testing.^48^ Evaluation of HPV sensitivity in self‐collected samples using HerSwab and physician‐collected samples did not result in any significant difference^45^ implying that this test is equally effective. For example, a survey done in Canada to assess the predictive value of HerSwab HPV collected samples to physician‐collected samples revealed that HPV was detected in 329 women (47%) with HerSwab and 327 (46.7%) with physician sampling.^35^ HPV16/18 for HerSwab and Physician collected samples were 43.7% and 43.8%, respectively. Sadly, some authors from the United States and Zimbabwe ascertained that this method of screening has not been evaluated and approved by the FDA in the US and other developing countries.^41^

Some authors have reported that countries like Japan that have not adopted HPV self‐sampling method have commenced clinical trial studies to evaluate its effectiveness and subsequent adoption.^49^ It was also suggested the need to evaluate newly developed vaginal self‐screening devices to increase their accessibility by women and also to identify and solve barriers that may hinder their implementation.^46^ More randomized control trial studies are therefore needed across various countries to evaluate the effectiveness, acceptability, and anticipated and unanticipated challenges of implementing HerSwab tests nationwide. This new technology should be evaluated in less advanced countries such as those in Sub‐Saharan Africa (SSA) to detect how well it could be introduced and well implemented in these countries. Evaluation could also include personal experiences and stories of women who have used the HerSwab self‐sampling method on how they obtained the test kits, storage, transportation, communication of results, and follow‐up if any, and all of which could affect acceptance of this new method.

## 6 ASSESSMENT OF HERSWAB DIAGNOSTIC TEST

As HerSwab is a newly designed diagnostic test, there is a need for assessment and evaluation of this test kit to ensure its adoption in other settings where it is not yet approved so that it can be patronized by females worldwide, especially in less advanced countries. This could be done through storytelling and assessing lived experiences of women in other parts of the world who have adopted this innovation. The users can contribute to its evaluation, and to make changes where appropriate for easy adoption, implementation, and patronization by women worldwide to help reduce CC. This is because previous studies have suggested the use of stories to improve behavioral change.^50^, ^51^ Another innovative way of assessing HerSwabs for HPV screening is to ensure its effectiveness and uptake is through prototyping which is an active component of design thinking in healthcare management. With this, HerSwabs diagnostic test could be piloted in some facilities in countries where it has not been introduced. This would contribute to its refinement, and address challenges and feedback for better patronization and acceptance.

Another component of design thinking that could help in the assessment of the HerSwab diagnostic test kit is the User/Need assessment.^4^, ^29^ With this, observational surveys and interviews could be done in countries where HerSwabs for HPV screening and CC are done in to be able to appropriately generalize it to other countries yet to adopt this service. Greater adoption would help reduce the global burden of CC. Assessment of HerSwab diagnostic test could also be achieved by employing the use of mobile health (mhealth) as other scholars have identified a positive correlation between mobile health and cervical cancer screening uptake.^52^, ^53^, ^54^ This could be achieved by involving a multidisciplinary team (physicians, nurses, laboratory technicians, IT specialists, etc) to help in designing apps/tools on mobile phones of women who have or are interested in utilizing this method. The technology could help women know the challenges and recommendations on ways to improve how the kit is used as it could increase rates of patronization to help in early detection and early detection of CC.

Online educational tools could also be developed and made accessible to women worldwide to help direct them on the effective ways of sample collection, storage, and transport to obtain accurate results. Finally, the previously developed diagnostic test for HPV (Pap Test and HPV DNA) has faced challenges such as cueing which could result in stress and burnout among the healthcare professionals conducting the screening.^55^ HerSwab HPV self‐sampling test will not only improve the health of populations, and patients, and reduce the per capita cost (triple aim), but could go a long way to reduce stress and burnout among healthcare professionals conducting the screening worldwide (Quadruple aim) in various facilities worldwide^56^, ^57^ as samples are self‐collected.

In conclusion, cervical cancer is one of the most frequently diagnosed cancers among women globally, and it is associated with HPV which is sexually transmitted. Recent studies have been focused on cervical cancer prevention, most especially cervical cancer screening. However, the screening for cervical cancer has faced varied challenges including poor patronization as its introduction. It is therefore imperative for innovative strategies such as self‐sampling HPV screening methods (HerSwab) to be introduced, implemented, and evaluated to help improve the uptake of cervical cancer screening to reduce the incidence and late diagnosis of cervical cancer. Even though the HerSwab HPV sampling method has been introduced and approved in some countries like Canada it is not widely available in other countries most especially in less developed countries. Future studies should therefore be geared toward strategies for worldwide approval of self‐sampling HPV methods and their acceptability by women.

## AUTHOR CONTRIBUTIONS

**Evans Appiah Osei:** Conceptualization (equal); formal analysis (equal); investigation (equal); methodology (equal); validation (equal); visualization (equal); writing – original draft (equal); writing – review and editing (equal).

## CONFLICT OF INTEREST STATEMENT

There is no competing interests for this study.

## Data Availability

All supporting data have been made available and have been uploaded with the manuscript.
